# The causal effect of serum micronutrients on malignant kidney neoplasm in European descent

**DOI:** 10.3389/fonc.2023.1191825

**Published:** 2023-08-16

**Authors:** Pengfei Qiao, Zhentao Tian

**Affiliations:** ^1^ The Department of Urology Surgery, First Teaching Hospital of Tianjin University of Traditional Chinese Medicine, Tianjin, China; ^2^ National Clinical Research Center for Chinese Medicine Acupuncture and Moxibustion, Tianjin, China

**Keywords:** micronutrients, causal estimation, cancer prevention, Mendelian randomization analysis, kidney neoplasms

## Abstract

**Purpose:**

Observational studies have revealed that serum minerals and vitamins are associated with cancer. However, the causal relationships between serum minerals and vitamins and renal malignancies remain unclear.

**Methods:**

Mendelian randomization (MR) was used for causal estimation. Single nucleotide polymorphisms (SNPs) for serum minerals and vitamins were obtained from published genome-wide association studies (GWAS). GWAS for malignant kidney neoplasm was obtained from the FinnGen consortium. Methods of inverse variance weighted (IVW), MR-Egger, and weighted median were carried out for causal inference. F-statistic was calculated to ensure a robust instrumental variable. Cochran’s Q statistics was applied to calculate heterogeneity. MR-Egger regression, MR-pleiotropy residual sum and outlier methods (MR-PRESSO) methods were used to perform pleiotropy analysis. Meanwhile, confounding factors were considered to determine whether causal inference would be biased.

**Results:**

Eight different micronutrients were included (zinc, iron, magnesium, calcium, copper, selenium, phosphate, and vitamin B12). After MR analysis, we found a protective effect of serum zinc against malignant kidney neoplasm (IVW: odds ratios (ORs), 0.86; 95% confidence interval (95% CI), 0.78–0.94; *p*, 0.0016; MR-Egger: OR, 0.80; 95% CI, 0.64–0.97; *p*, 0.052; weighted median: OR, 0.85; 95% CI, 0.75–0.96; *p*, 0.011). Causal relationships between other micronutrients and malignant kidney neoplasm were not obtained. No heterogeneity and pleiotropy were detected, while causality was not biased by confounding factors.

**Conclusion:**

We considered that serum zinc exerted a protective effect against malignant kidney neoplasm. In clinical practice, for people with high malignant kidney neoplasm risk, an oral zinc supplementation might play a role in a potential therapeutic target.

## Background

In 2020, there are 431,288 new cases of kidney cancer and 179,368 new cancer deaths in 185 countries (1). In Europe, the number of deaths from kidney cancer exceeds 50,000 in 2018 (2). Screening and early detection had been identified as a top priority in kidney cancer research (3). Significant correlation was found between early diagnosis of kidney cancer and patient survival. Cancer-specific 5-year survival rates for stage I and IV kidney cancer patients were 83% and 6%, respectively. However, approximately 20% of patients with kidney cancer demonstrated signs of metastasis in Europe (4, 5).

In spite of the current advanced medical technology, the incidence of kidney cancer is still increasing. Modifiable risk factors for kidney cancer, such as lifestyle habits (smoking, obesity, hypertension, excessive alcohol intake, and diet), were increasingly being emphasized (6). Targeting modifiable risk factors has become a strategy to reduce the incidence of cancer (7). Associations between dietary nutrients and cancer have been reported in numerous studies. A randomized controlled trial by François et al. concluded that men with standard PSA who received the supplement (zinc, selenium, beta-carotene, vitamin E, and vitamin C) demonstrated a statistically significant decline in prostate cancer incidence and were statistically significant (hazard ratio, 0.52; 95% CI, 0.29–0.92) (8). Ray et al. examined zinc levels in hair to explore its role in esophageal cancer and concluded that zinc deficiency led to an increased risk in developing esophageal cancer (9). A study by Khoshdel et al. founded that there was a lower plasma zinc concentration in the colorectal cancer group compared to the control group (10). Association between serum micronutrients and renal malignancies was also revealed. Serum folate and vitamin B12 levels were associated with decreased risk of renal cell carcinoma (11) Research by Panaiyadiyan et al. found a significantly higher serum concentration of arsenic, copper, manganese, cadmium, lead, and mercury while a lower serum concentration of selenium in patients with renal cell carcinoma compared to controls (12). Xu et al. revealed that low vitamin D status was associated with an increased risk of renal cell carcinoma (13). However, it is uncertain whether there is a causal relationship between serum micronutrients and renal malignancies.

**Figure 1:**
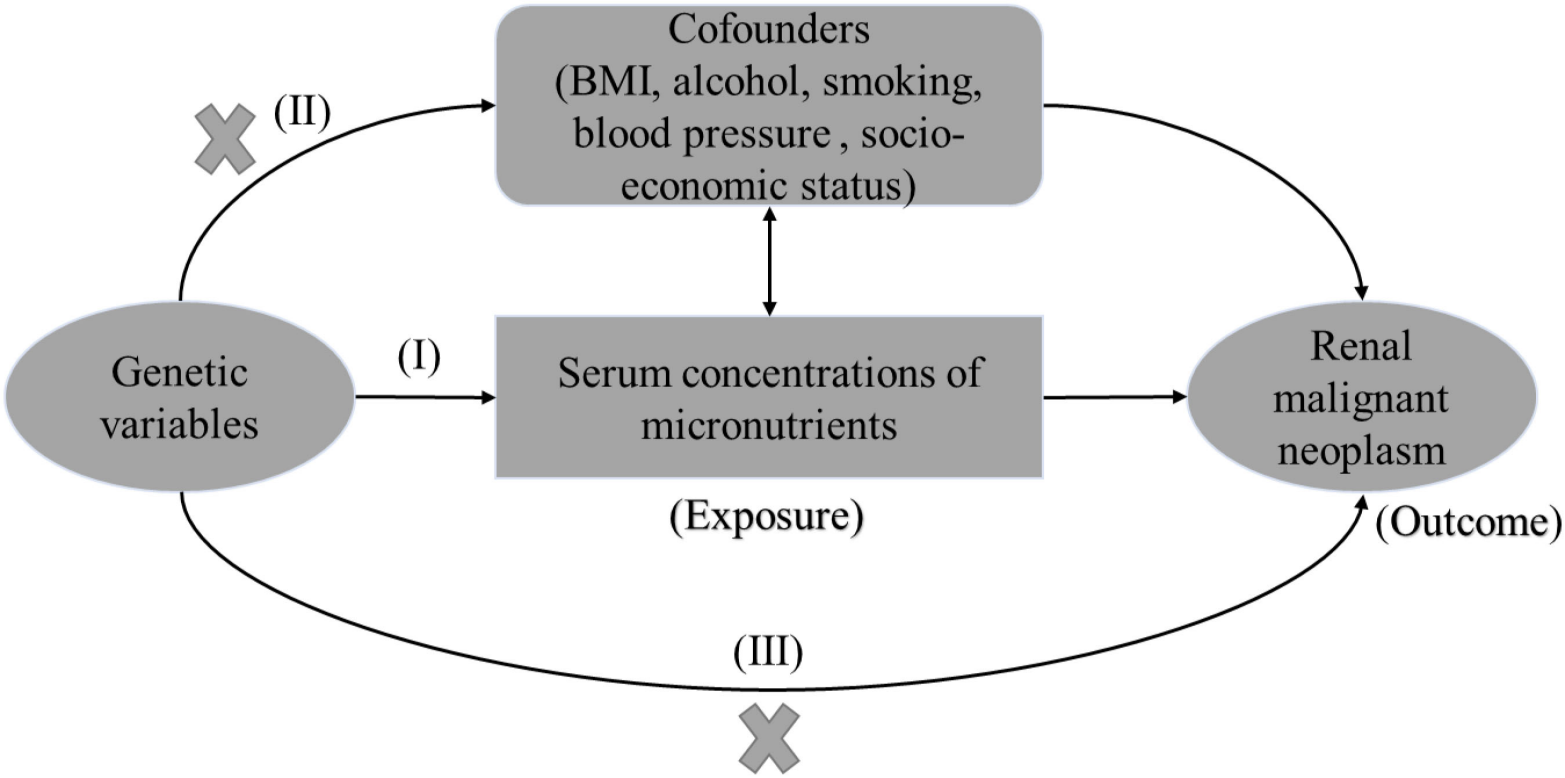
The three mendelian assumptions in the study. The instrumental variables should: **(I)** be robustly associated with serum micronutrients (exposure of interest); **(II)** be independent of BMI, alcohol, smoking, blood pressure (confounders); and **(III)** have no direct association with the renal malignant neoplasm (outcome of interest), but affect outcome exclusively through serum micronutrients. BMI, body mass index.

With the increasing number of large-scale genome-wide association studies (GWAS), Mendelian randomization (MR) has been used to make causal inference in different phenotypes. By using genetic variants as instrumental variables, MR can overcome the interference of reverse causality and minimize confounding factors, but still suffer from residual confounding (14). The MR assumptions are presented in **Figure 1**.

In the present study, we used genetically proxied serum micronutrients and malignant kidney neoplasm to explore their causal relationship using a two-sample MR approach. Meanwhile, we evaluated whether potential confounders induced bias in causal inference. As mentioned earlier, smoking, alcohol abuse, obesity, and blood pressure are modifiable factors for renal malignancy. Meanwhile, serum micronutrients are related to those factors (15–18). Therefore, we took smoking, alcohol abuse, body mass index (BMI), and blood pressure as confounding factors. Generally, adherence to a healthier lifestyle is associated with higher educational and socio-economic level of the family. Thus, household socio-economic status is also a potential confounding factor. Within our knowledge, studies on the causal relationship between serum micronutrients and renal malignancies are still limited, and we comprehensively elucidate the causal relationship through MR analysis on our research.

## Methods

### The acquisition of exposure GWAS data

GWAS data for serum zinc and copper were derived from Ng et al.; micronutrients were assayed in a cohort of 949 individuals using mass spectrometry. DNA samples were genotyped on Infinium Omni Express magnetic bead microarrays and extrapolated from 1000 Genome Project reference plates (19). We performed further analyses for the original data because the possibility of linkage disequilibrium (LD) of individual single nucleotide polymorphism (SNP) loci was not considered; LD of r^2 =^ 0.001 and physical distance kb=10,000 were set to minimize linkage disequilibrium. GWAS data for serum iron, magnesium, calcium, selenium, phosphate, and vitamin B12 were obtained from Tsilidis et al. This study was conducted with strict quality control to avoid the presence of collision bias. SNPs that were related to the concentrations of serum micronutrients at a genome-wide significance level (*p*<5×10^−8^) and were not in linkage disequilibrium (LD of r^2 =^ 0.01) were selected (20).

### The retrieval of the outcome GWAS data

GWAS data for malignant kidney neoplasm was obtained from the FinnGen consortium (https://www.finngen.fi/fi), consisting of 1,631 patients and 307,523 controls. The diagnosis criteria for of kidney cancer were ICD-O-3 code. We extracted exposure-related SNPs in the outcome, and if SNPs that were significantly associated with serum micronutrients were also related to renal malignancy (*p*<0.05) were excluded.

### The obtainment of GWAS data on confounding factors

When performing MR analysis, the causal relationship between exposure and outcome cannot be mediated by confounders; therefore, a causal relationship between exposure and confounders must be excluded. Smoking, alcohol abuse, BMI, blood pressure (systolic, diastolic), and socio-economic status (average total household income before tax, full-time or part-time education) were common confounders. The GWAS data for these confounders were obtained from OpenGWAS database (21), and the specific information is presented in **Table 1**.

**Table 1:** Details of confounders and casual inference between genetically predicted serum zinc and cofounders.

Trait	Consortium	Sample size	OR	95%CI	*p*
**Ever smoked**	MRC-IEU	461,066	1.00	(0.99,1.00)	0.38
**Cigarettes smoked per day**	GSCAN	249,752	0.99	(0.98,1.00)	0.44
**smoking initiation**	GSCAN	607,291	1.00	(0.99,1.01)	0.44
**Alcohol intake frequency**	MRC-IEU	462,346	0.99	(0.99,1.00)	0.95
**BMI**	MRC-IEU	461,460	1.00	(0.99,1.01)	0.07
**Diastolic blood pressure**	MRC-IEU	39,749	0.99	(0.98,1.01)	0.76
**Systolic blood pressure**	MRC-IEU	436,419	0.99	(0.99,1.00)	0.15
**Average total household income before tax**	MRC-IEU	397,751	1.00	(0.99,1.01)	0.17
**Full-time or part-time education**	NA	688,99	0.99	(0.99,1.00)	0.08

BMI, body mass index; OR, odds ratio; CI, confidence interval.

### Statistical analysis

In our study, we used the inverse variance weighted (IVW), MR-Egger, and weighted median methods for causal inference. Different methods were used because these methods had different conditional assumptions. The IVW approach could be performed either by random effects model or fixed effects model. *p*<0.05 of Cochran’s Q statistics was used as a threshold to decide whether a random effects model or a fixed effects model was chosen (22). The IVW approach, which performed a meta-analysis of the Wald ratios of individual SNPs, assumed that the genetic variants could only influence the outcomes through the exposure of interest and not through any other pathways (23). MR-Egger and weighted median could complement the IVW approach in a broader scenario. In the sensitivity analysis, we used Cochran’s Q statistics to detect heterogeneity. MR-Egger regression, MR-pleiotropy residual sum and outlier methods (MR-PRESSO) were employed to detect pleiotropy, which referred to the fact that proxied SNPs led to the occurrence of outcome by other pathways. The intercept in MR-Egger regression approach was considered as a sign of the presence of pleiotropy, but *p*<0.05 was taken as a statistical criterion. MR-PRESSO could detect the presence of outlier SNPs and filter them out, and provide detection of statistical differences in causal assessments before and after the removal of outliers. The leave-one-out method was adopted to examine whether the causal assessment would be biased by a particular SNP. R^2^ was taken to evaluate the proportion of variance explained by SNPs (R^2 =^ 2 × (1 − MAF) × MAF × (β)^2^). The F-statistic was adopted to calculate the strength of the instrumental variable (F = β^2^/se^2^). F>10 was usually considered as a strong instrumental variable. β was the coefficient for the SNP, MAF was the minor allele frequency, and se was the standard error of coefficient. All MR analyses were performed in R (version 4.2.1) with the help of the R packages “TwoSampleMR” (21) and “MRPRESSO” (24).

## Results

### Causal effect from serum zinc on malignant kidney neoplasm

**Figure 2:**
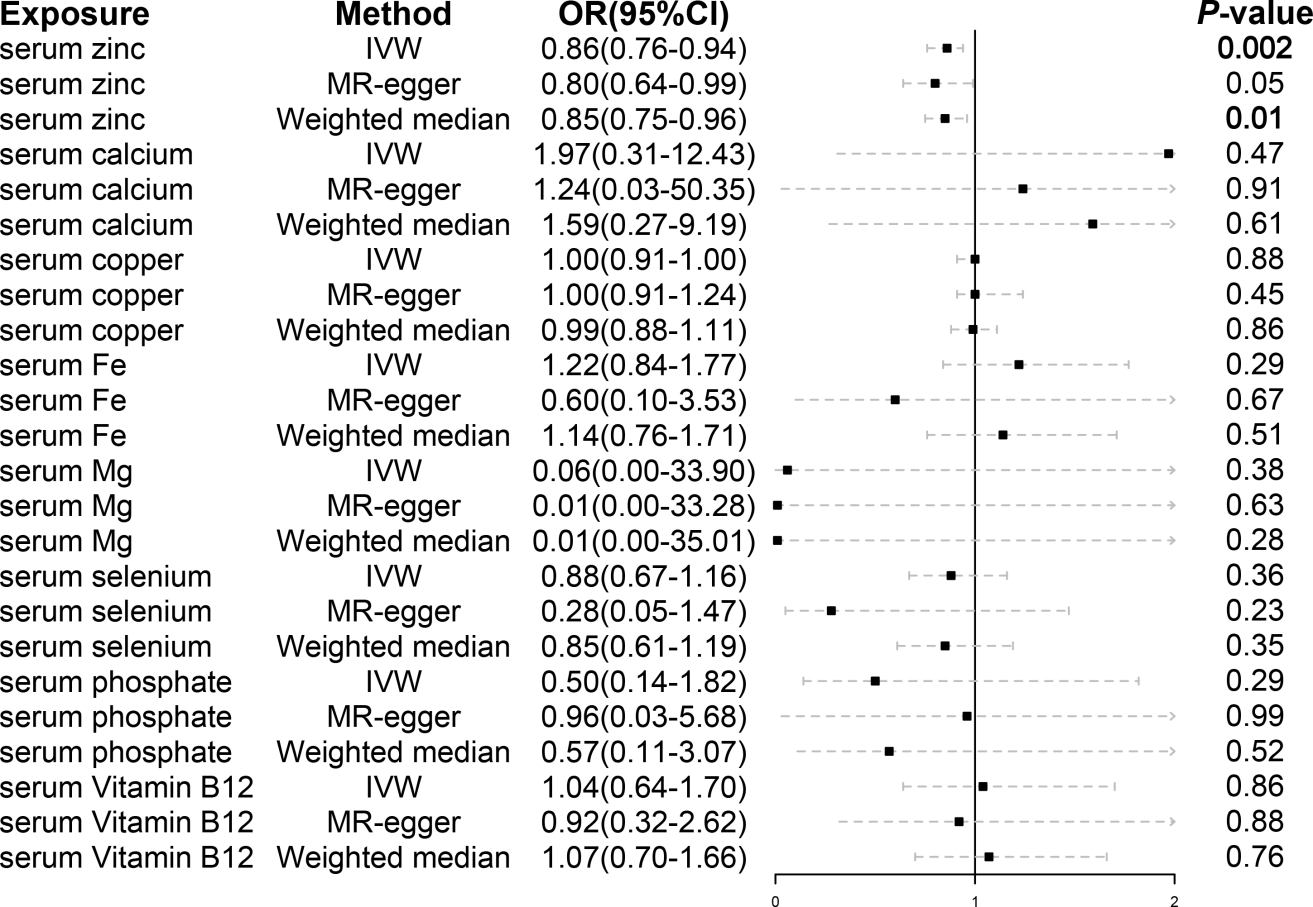
The casual inference of serum micronutrients on malignant kidney neoplasm using IVW, MR-Egger, and weighted median methods. IVW, inverse variance weighted; OR, odds ratio; CI, confidence interval.

**Figure 3:**
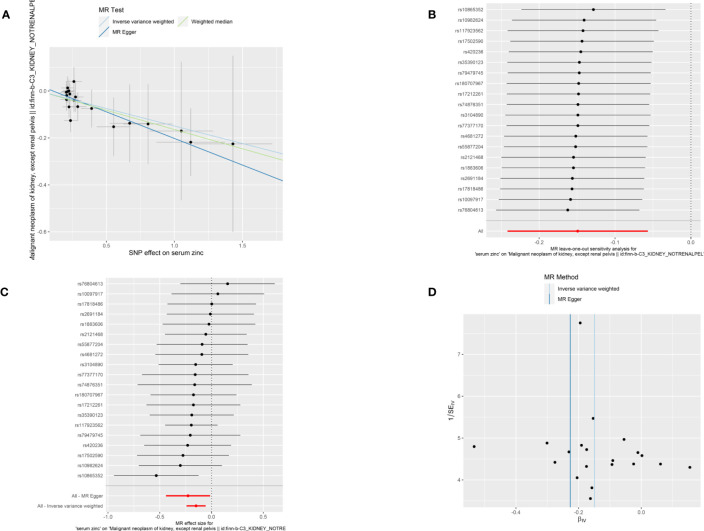
Visualization of causal inference from serum zinc on malignant kidney neoplasm. **(A)** MR-Egger regression slope. **(B)** Leave one out analysis. **(C)** Forest plot. **(D)** The funnel plot.

A total of 21 SNPs associated with serum zinc were included for causal estimation. The SNPs explained 2.0%–2.7% of the variance in circulating zinc concentration. The mean value of F-statistic was 21.7, ranged from 19.53 to 27.77 (**Supplementary Table S1**). In the merge process, rs2635551 was not found in the outcome. In MR-PRESSO, no outliers were detected, and the *p-*value for Global Test was 0.979. A total of 20 SNPs were finally included in the final MR causal estimation. Using the IVW approach, we detected a statistically significant causal relationship between serum zinc and malignant kidney neoplasm (OR, 0.86; 95% CI, 0.78–0.94; *p*, 0.0016). The result was also remarkable in weighted median (OR, 0.85; 95% CI, 0.75–0.96; *p*, 0.011). However, to our disappointment, the method of MR-Egger did not produce a causal relationship (OR, 0.80; 95% CI, 0.64–0.97; *p*, 0.052) (**Figure 2**). Heterogeneity was not detected with methods of IVW, and the *p*-value of Cochran’s Q statistics were 0.98. In the test of pleiotropy, the intercept was 0.024, but it was not statistically significant (*p*, 0.44) (**Table 2**). MR regression slope is displayed in **Figure 3A**. The result of leave-one-out also indicated that none of the SNPs had a distinct effect on causal inference, and this relationship exhibited robustness (**Figure 3B**). Forest plot could display the effect of individual SNP on causal assessment (**Figure 3C**). Funnel plot demonstrated that the distribution of SNPs tended to be balanced (**Figure 3D**).

**Table 2:** Characteristics of the GWAS summary data for serum micronutrients.

Exposure		Outcome				
Trait	PMID	nSNP^1^	nSNP^2^	*P* (mr_presso)	*P* (pleiotropy)	*P* (heterogeneity)
Serum zinc	26025379	21	20	0.21	0.44	0.98
Serum copper	26025379	25	25	0.55	0.33	0.54
Serum iron	33740060	3	3	NA	0.57	0.42
Serum magnesium	33740060	6	5	0.57	0.82	0.53
Serum calcium	33740060	7	7	0.25	0.78	0.14
Serum selenium	33740060	7	5	0.47	0.26	0.41
Serum phosphate	33740060	5	5	0.35	0.88	0.33
Serum vitamin B12	33740060	9	9	0.06	0.80	0.03

nSNP^1^, the total SNPs after the screen using the p-value, physical distance (kb > 10,000), and linkage disequilibrium (R^2^ < 0.001); nSNP^2^, the total SNPs after the removal of incompatible, palindromic, and outlier SNPs; P (mr_presso): The P for the testing of outlier SNPs.

### Causal effect from other serum micronutrients on malignant kidney neoplasm

A total of 9, 7, 25, 3, 6, 5, and 7 SNPs of serum vitamin B12, calcium, copper, iron, magnesium, phosphate, and selenium, respectively, were included for causal estimation. In the merge process, the genetically proxied rs7965584 for magnesium and rs6586282 and rs1789953 for selenium were not found in outcome. All the SNPs were robust genetic variants. In MR-PRESSO, no outliers were detected. After MR analysis, none of the above serum micronutrients had causal associations with malignant kidney neoplasm (**Figure 2**). Pleiotropy was not found (**Table 2**).

### Causal effect from serum zinc on potential cofounding factors

To exclude the bias of confounders in the causal inference from serum zinc on malignant kidney neoplasm, we evaluated potential confounders such as smoking, alcohol consumption, obesity, and blood pressure. We obtained GWAS data for risk factors: smoking (ever smoked, smoking initiation, and cigarettes smoked per day), alcohol abuse (alcohol intake frequency), blood pressure (systolic blood pressure and diastolic blood pressure), obesity (BMI), and socio-economic status (average total household income before tax and full-time or part-time education). The IVW approach was taken to assess the causal relationship between serum zinc and cofounding factors. As shown in the **Table 1**, causal relationships were not observed.

## Discussion

In the present study, we used published GWAS data to infer the causal relationship between serum micronutrients and malignant kidney neoplasm. We found that serum zinc reduced the risk of malignant kidney neoplasm causally. Furthermore, the causal inference was not biased by potential confounding factors. However, we did not obtain a causal relationship between other serum micronutrients and renal malignancies.

As a common antioxidant, the anticancer effect of zinc has been widely studied. Many observational studies have explored the relationship between zinc and kidney cancer, but no consensus has been reached. Wang et al. reveal that zinc played an important role in promoting the development of kidney cancer (25). Zhang et al. suggested that an above median zinc was associated with a reduced risk of kidney tumors (26). We assumed that different geographical locations and various dietary habits led to distinct food sources of zinc, so we could not judge whether other substances in food would influence the results. In addition, the adjustment for confounding factors varied. For example, high dietary intake of zinc increased the risk of stone (27), and renal stone was a risk factor for malignant kidney neoplasm (28); therefore, it was possible that the presence of renal stones also mediated the association between zinc and malignant kidney neoplasm.

The anticancer effect of zinc was mainly attributed to its antioxidant properties. Under homeostatic conditions, the body produced reactive oxygen species (ROS), which were involved in many biological processes (cell differentiation, apoptosis) (29, 30). Due to the high metabolic demand of tumors, tumor tissues produced large amounts of ROS, which promoted tumor growth by causing DNA damage and inducing gene mutations (31, 32). The antioxidant effect of zinc then exerted a very good role in inhibiting tumor growth. The antioxidant of zinc was primarily due to its ability to act as a component of superoxide dismutase, preventing the production of free radicals and their derivatives (33, 34). In addition, zinc acted as an antagonist of iron and copper involved in lipid peroxidation, thus reducing the production of free radicals (35, 36).

Meanwhile, zinc could also have an impact on the immune system. The impact of zinc on the immune system was primarily through the Th lymph nodes. Zinc deficiency led to an imbalance of Th1 and Th2, which contributed to a dysfunctional immune response (37, 38). Zinc supplementation slowed this imbalance by increasing IFN-γ (Th1 inducer). Additionally, IFN-γ itself had immunomodulatory and antitumor effects (39). Zinc deficiency impaired granulocyte recruitment, ROS production, chemotaxis, and phagocytosis (40, 41). Adherence of mononuclear cells to epithelial cells also occurred in a zinc-dependent manner (42).

Since zinc got involved in the formation of zinc fingers, the latter could perform a significant role in regulating DNA replication, transcription, transformation, and repair due to its high selectivity and capacity to bind to DNA, RNA, and proteins (43, 44). Furthermore, zinc ensured the clearance of mutated or damaged forms that could lead to cancer by participating in the regulation of apoptosis (45). Endogenous zinc was also indispensable in the induction of autophagy under conditions of oxidative stress.

A couple of advantages were present in our study. First, we observed a protective effect of serum zinc concentration against malignant kidney neoplasm, so that zinc supplementation might be a potential clinical option for clinically high-risk population. However, more studies are needed to verify the conclusion. Second, our study used publicly available GWAS data to make causal inferences, and the statistical strength was higher due to the large sample size. Several drawbacks were also present in our study. First, we were not able to perform subtype analysis of malignant kidney neoplasm. The MR analysis was performed using summary GWAS data, and individual-level data were not available. The cancer subtype in each individual were also unknown. Second, our analysis was limited to an ancestral European sample, and it remained to be verified whether the same results would be obtained in other populations. Third, our study was only a basic theoretical study, and more animal experiments and cohort studies were needed to confirm the conclusions, before applied into clinical practice. Fourth, a causal relationship between other serum malignancies and renal malignancies were not obtained. Possible reasons were as follows: (i) the study population in this study was mainly of European origin, and it was likely that the sensitivity of renal malignancies to serum micronutrients differed in different populations; (ii) causal associations were probably not present between other micronutrients and renal malignancies; it was probable that previous observational studies have not controlled for confounding factors better; and (iii) phenotypes were defined differently in different studies.

## Conclusion

In conclusion, we obtained that genetically proxied serum zinc reduced the risk of malignant kidney neoplasm causally. Therefore, zinc might play a role in a potential therapeutic target for renal malignancies.

## Data availability statement

The original contributions presented in the study are included in the article/**Supplementary Material**. Further inquiries can be directed to the corresponding author.

## Author contributions

All authors listed have made a substantial, direct, and intellectual contribution to the work and approved it for publication.

## Supplementary material

Additional file 1 Detailed information of the proxied SNPs of serum zinc concentration.
